# Occupational Well-Being in Beginning Early Childhood Educators of Hong Kong and the Prediction of Job-Related Factors: Variable-Centered and Person-Centered Approaches

**DOI:** 10.3389/fpsyg.2021.746123

**Published:** 2021-11-25

**Authors:** Jian-Bin Li, An Yang, Rui Zhang, Tuen Yung Leung, Zhouxing Li

**Affiliations:** ^1^The Education University of Hong Kong, Tai Po, Hong Kong SAR, China; ^2^Guangdong University of Foreign Studies, Guangzhou, China; ^3^Department of Psychology, Guangzhou University, Guangzhou, China; ^4^University College London, London, United Kingdom; ^5^Caritas Institute of Higher Education, Hong Kong, Hong Kong SAR, China; ^6^Guangdong University of Education, Guangzhou, China

**Keywords:** professional well-being, job demands, job resources, transition, JD-R model, person-oriented approach, variable-oriented approach, kindergarten teachers

## Abstract

The occupational well-being (OWB) of early childhood educators (ECEs) play a crucial role in their job performance, the development of a child, and the operation of early childhood education sectors. OWB of ECEs has been an increasing concern in recent years and this concern might be particularly salient for beginning ECEs given the multiple adaptive changes and challenges they encounter during the transition from training to teaching. However, research on the beginning ECEs’ OWB has been scarce. In this study, we employed both variable-centered and person-centered approaches to explore OWB and examined job demands and resources as predictors in 117 Hong Kong beginning ECEs (113 females, *M*_*age*_ = 21.71 years). They first reported job demands (i.e., class size, working hours, dealing with children with special education needs) and job resources (i.e., salary and job support) at the end of the first month of the fall semester upon working as in-service teachers. They then reported on four OWB variables (i.e., job satisfaction, work engagement, job stress, and job burnout) at the beginning of the spring semester. Results of the variable-centered analysis revealed that beginning ECEs reported medium or above-medium levels on the positive OWB indicators and medium or below-medium levels on the negative OWB indicators. Regression analysis found that despite some exceptions, job demands and job resources negatively (positively) and positively (negatively) predicted positive (negative) OWB indicators, respectively. Results of person-centered analysis suggested that the complex pattern of different OWB indicators could be categorized into two OWB profiles (*medium well-being* vs. *relatively weak well-being – emotional exhaustion*). Results of regression analysis showed that beginning ECEs with higher job demands were less likely, whereas those with more job resources were more likely, to be assigned to the medium well-being profile. These results inform *which* leverage points could be targeted to enhance a specific OWB indicator and identify *who* would be in dire need to enhance their OWB at the very beginning of their teaching career.

## Introduction

There is an increasing concern for the occupational well-being (OWB) of early childhood educators (ECEs) in recent years around the globe (Hall-Kenyon et al., 2014; Cumming, 2017). This is all the more important because a positive OWB is associated with a range of desirable consequences, such as better job performance, more capacity to provide quality education and supportive classroom climate, and stronger intention to stay in the early childhood education (ECE) sector (Jeon et al., 2019; Penttinen et al., 2020; Schaack et al., 2020; Sönmez and Betül Kolaşınlı, 2020; Li et al., 2021), whereas the nature of the job itself (e.g., interactions with children, handling challenging behavior of children) entails numerous challenges and demands that often put OWB of ECEs at risk (Whitaker et al., 2015 Gu et al., 2020). Prior research has examined OWB of ECEs and its associated predictors, revealing that sufficient job resources (e.g., job support) and few job demands (e.g., long working hours) are linked with a positive OWB (Cheng and Chen, 2011; Li et al., 2020; Schaack et al., 2020). Although the existing literature is informative, most of them are variable-centered and may not reflect the full picture of OWB of ECEs because, in reality, employees may simultaneously endorse seemingly contradictory measures of OWB, such as burnout and engagement (Moeller et al., 2018; Salmela-Aro et al., 2019). Besides, most of the existing studies are conducted among veteran ECEs or ECEs with at least several years of teaching experience, but much less is focused on beginning ECEs. Understanding the OWB of beginning ECEs is important because novice teachers are likely to experience “adaptive shocks” that may be threats to their OWB during the transition from training to teaching (Chaaban and Du, 2017). For instance, prior research has suggested that compared to veteran teachers, beginning teachers experience more problems in classroom management, are less satisfied with the salary, and may over time want to leave the job (Chaaban and Du, 2017; Jones et al., 2019; Schaack et al., 2020). To bridge the said gaps, the aim of this study is twofold. First, we employ both variable and person-centered approaches to examine OWB of beginning ECEs. Second, we examine the extent to which the job-related factors relevant to the ECE settings predict subsequent OWB from a job demands-resources (JD-R) perspective (Bakker and Demerouti, 2014). The results are expected to contribute to the literature of OWB of ECEs.

### Early Childhood Educators in Hong Kong and Beginning Early Childhood Educators

This study is specifically focused on beginning ECEs in Hong Kong. Over the past two decades, the Hong Kong government has implemented several reformations in the ECE system to develop one of the best, most competitive kindergarten education policies in the world, such as increasing the financial investment in kindergarten education (c.f. Wong and Rao, 2015). In Hong Kong, there are more than 1,000 kindergartens and kindergartens-cum-childcare centers (Education Bureau, 2021), and nearly all the children aged 3 to 6 attend preschool programs even though ECE is not compulsory (Audit Commission, 2013). According to Wong and Rao (2015), an important reason for such a high enrollment rate in ECE is because the education systems in Hong Kong is highly competitive, wherein examinations are heavily emphasized, and parents typically perceive ECE as a means to prepare their children to be ready for a primary school in an early stage. Other reasons also include a large number of dual-earning families, the extensive and efficient public transport system, and above all, the implementation of various funding schemes which ensure that no child is deprived of preschool education because of financial issues (Rao, 2010).

To promote learning diversity, all-round development, and better alignment with primary and secondary education of children, the Education Bureau has implemented the Free Quality Kindergarten Education Schemes since 2017 to provide even more affordable and better quality of education to children through improving the school environment and enhancing the professionalism of teachers (Education Bureau, 2017). As of 2021, about 73% of the kindergartens in Hong Kong have joined the scheme (Education Bureau, 2021). Although the professional skills and job performance of ECEs are greatly emphasized in Hong Kong, their OWB has received much less attention. This is concerning as a recent survey conducted with 1,255 Hong Kong ECEs (referred to teachers in kindergartens-cum-childcare centers) found that many ECEs had experienced alarming issues [e.g., 82% reporting more than 50 working hours per week; 53% reporting increase in administrative work since the implementation of the scheme; 65% reporting the need to deal with children with special education needs (SEN); and 71% reporting incommensurate salary]. Meanwhile, many of them hoped to have more support, resources, staffing, and commensurate salary (Hong Kong Federation of Education Workers, 2019).

This study is contextualized in the developmental stage where young people transition from higher education to work. To register as a kindergarten teacher in Hong Kong, students need to complete a recognized training program, such as a 2-year full-time higher diploma in ECE or a four or 5-year full-time Bachelor of Education in ECE. Graduates of the higher diploma and Bachelor of Education (full-time) typically fall within the emerging adulthood (aged 18–25 years, Arnett, 2000) as these students are usually admitted to university right after high school. These fresh graduates not only need to solve multiple developmental tasks relevant to emerging adulthood, such as exploring adult identity, becoming financially independent, and developing romantic relationships (Arnett, 2000), but they are also required to navigate many adaptive changes after serving as beginning teachers, such as role transition, inexperience, and unfamiliarity with the new organization (Chaaban and Du, 2017; Zhou et al., 2020). As such, the first year of teaching is often filled with struggling and successfully handling the adaptive changes would result in enjoyment, engagement, and satisfaction, whereas failure to do so leads to negative emotions, self-doubts, dissatisfaction, and burnout (Tait, 2008) and further causes cascade of inefficacy and attrition within the first few years of teaching (Tiplic et al., 2015; Harmsen et al., 2018). Taken together, understanding OWB among Hong Kong beginning ECEs is paramount and necessary.

### Occupational Well-Being Among Early Childhood Educators

The occupational well-being of teachers can be defined as the “positive evaluation of various aspects of one’s job, including affective, motivational, behavioral, cognitive, and psychosomatic dimensions” (Van Horn et al., 2004, p. 366). Although little research has exhausted occupational well-being elements of ECEs so far, existing literature has commonly agreed that OWB of ECEs is a broad construct involving both positive and negative indicators and that *job satisfaction*, *work engagement*, *job stress*, and *job burnout* could be the four most representative elements (Hall-Kenyon et al., 2014; Cumming, 2017; Penttinen et al., 2020; Peele and Wolf, 2021). These elements are consistent with the core ideas of positive psychology that the optimal well-being of an individual should be reflected by both the absence of illness and the presence of virtues (Seligman and Csikszentmihalyi, 2000). These elements also align with the two approaches of well-being: the hedonic approach which focuses on pleasure attainment and pain avoidance and the eudaimonic approach which focuses on the degree to which a person is fully functioning (Ryan and Deci, 2001). ECEs with a positive OWB are those who score high on positive and low on negative indicators. By contrast, ECEs with a negative OWB refer to those who score low on positive and high on negative indicators.

In this study, *job satisfaction* refers to the evaluative judgments that teachers make about their work (Corbell et al., 2010). Job satisfaction of teachers may result from both intrinsic and extrinsic sources, but researchers suggest that compared to measuring intrinsic and extrinsic satisfaction separately, an overall evaluation of job satisfaction may capture a more objective sense of satisfaction because this would reduce the measurement bias due to differential evaluation of the same contextual factor at various time points (Chaaban and Du, 2017). Regarding *work engagement*, it refers to a positive, fulfilling, and work-related state of mind featured by vigor, dedication, and absorption (Schaufeli et al., 2002). According to Schaufeli et al. (p. 74–75), *vigor* is featured by “high levels of energy and mental resilience while working, the willingness to invest effort in one’s work, and persistence even in the face of difficulties.” *Dedication* is featured by “a sense of significance, enthusiasm, inspiration, pride, and challenge.” *Absorption* is featured by “being fully concentrated and deeply engrossed in one’s work, whereby times passes quickly and one has difficulties with detaching oneself from work.” *Job stress* refers to work-related demands directly interfering with the effort of teachers, depleting time, and inducing unpleasant physical and emotional reactions (Blase, 1982). Job stress of ECEs may arise from a number of sources (e.g., time management, professional distress) and can be manifested in terms of emotional, behavioral, and somatic symptoms (Tsai et al., 2006). *Job burnout* is a concept relevant to job stress and it is a prolonged response to chronic emotional and interpersonal stressors on the job (Maslach et al., 2001). Job burnout is characterized by three key dimensions, namely, exhaustion, cynicism, and inefficacy. According to Maslach et al. (2001), *exhaustion* is the stress dimension of burnout and refers to feelings of being overextended and depleted emotional and physical resources of an individual. *Cynicism* (or depersonalization) is the interpersonal context dimension of burnout and refers to a negative, callous, or excessively detached response to various aspects of the job, and *reduced efficacy* (or low accomplishment) is the self-evaluation dimension of burnout and refers to feelings of incompetence and a lack of achievement and productivity at work. Noticeably, exhaustion and cynicism are the two opposite dimensions of vigor and dedication, respectively (Schaufeli et al., 2002). Burnout is often related to the intention of teachers to quit the job or the profession (Leung and Lee, 2006; Skaalvik and Skaalvik, 2011). Prior studies have suggested that these indicators are typically interrelated among one another (Schaufeli et al., 2006; Gu et al., 2020; Penttinen et al., 2020; Li et al., 2021). For instance, Yeşil Dağli (2012) found that job satisfaction was negatively related to job burnout among United States public kindergarten teachers. Similarly, a study by Li et al. (2020) conducted among mainland Chinese preschool teachers discovered that job satisfaction and job stress were related to a lower and higher likelihood of job burnout, respectively. Moreover, the study by Li et al. (2021) conducted among Hong Kong beginning kindergarten teachers found that work engagement was negatively related to job stress.

The aforementioned studies relied on a variable-centered approach. However, this approach does not entirely reflect the reality because employees may simultaneously report highly on both positive and negative OWB indicators (Moeller et al., 2018; Salmela-Aro et al., 2019). To reveal the complex pattern of many variables, the person-centered approach is suitable and may complement the variable-centered approach (Howard and Hoffman, 2018). According to Magnusson (2003), variable and person-centered approaches have their own focuses, assumptions, and statistical models. Specifically, the variable-centered approach focuses on the position of an individual on the level of a variable relative to other individuals. Starting from the perspective of stimulus-response or response-response, this approach considers that position of an individual on the level of one variable is related to his/her position on the level of another variable, assuming that “the interrelations among variables studied at the group level can be used to make inferences about how the variables function within individuals” (p. 14). The analytical models of this approach (e.g., correlation, regression) are well suited for questions pertaining to the description of individual variables and to the examination of the effects of predictors on the outcome variables. By contrast, the person-centered approach focuses on the identification of subgroups of individuals who function in a similar way while taking into account many variables simultaneously, assuming that a given population is heterogeneous and causing different subgroups based on similar configuration would emerge. The analytical models of this approach (e.g., latent profile analysis, LPA) are well suited for questions pertaining to group differences in the complex patterns of a construct indicated by many different variables. This approach does not assume that the interrelation between variables in one subgroup should hold for another subgroup. In sum, with a variable-centered approach, we may examine how well a person is doing relative to others in the sample on individual variables (e.g., job satisfaction) and the strength of associations among individual variables for the whole population. With a person-centered approach, we may determine whether the sample can be categorized into qualitatively distinct subgroups considering many individual variables simultaneously and examine what factors could distinguish the memberships of subgroups and how the subgroups differ in the outcomes.

A recent review summarized that in the organizational settings, the job, organizational attitudes, and behaviors of employees often display qualitatively distinct profiles comprised of different levels of indicators and that the use of a person-centered approach may nicely identify subpopulations with different configural profiles within a population to inform theoretical development (Spurk et al., 2020). For instance, Salmela-Aro et al. (2019) used LPA to explore OWB in terms of engagement and burnout among 149 Finnish high school teachers. They identified two profiles, namely, engaged-burnout and highly engaged. This suggests that while many teachers are fully engaged in their work, they may also report symptoms of burnout at the same time. In other words, not all teachers who score high on positive indicators (e.g., work engagement) would score low on negative indicators (e.g., job burnout). Using LPA in organizational research to disclose the OWB of employees, unfortunately, is still in its infancy (Spurk et al., 2020), let alone among beginning ECEs. Hence, the first aim of this study is to employ both variable and person-centered approaches to examine beginning the OWB of ECEs.

### The Role of Job Demands and Resources in the Occupational Well-Being of Beginning Early Childhood Educators

The job demands-resources (JD-R) model postulates that the OWB of employees is associated with two broad categories of factors, namely, job demands and job resources (Bakker and Demerouti, 2014). Job demands refer to various aspects of the job that require sustained physical and/or psychological effort that may lead to certain physiological and/or psychological costs, whereas job resources refer to various aspects of the job that help achieve the work goals, reduce job demands and their associated costs, and stimulate personal growth (Bakker and Demerouti, 2007). According to Bakker and Demerouti (2014), job demands exhaust the physical and psychological energy of employees and result in negative OWB. By contrast, job resources assist employees in navigating the job demands so that they can restore their energy, achieve desirable outcomes, and experience positive OWB.

Building upon the JD-R model (Bakker and Demerouti, 2014), the second aim of this study is to examine the role of different job demands and resources in predicting the OWB of Hong Kong beginning ECEs. According to the literature (Hall-Kenyon et al., 2014; Cumming, 2017; Hong Kong Federation of Education Workers, 2019; Ji and Yue, 2020; Li et al., 2020; Schaack et al., 2020; Zhou et al., 2020), the job demands that are common to ECEs, in particular, Hong Kong ECEs, include (but are not limited to) teaching a large sized class, handling challenging behaviors of SEN students, and working long hours, etc., whereas the job resources that are common to ECEs include (but are not limited to) receiving more support from supervisors and colleagues, being rewarded with commensurate salary, a good working climate, etc. Prior studies, albeit predominately cross-sectional in nature, found that ECEs encountering more job demands and possessing fewer job resources are more prone to experience negative OWB, while ECEs with fewer demands and more job resources tend to have a positive OWB (Gong et al., 2020; Li et al., 2020; Schaack et al., 2020).

Although little is known about the situation for beginning ECEs, in learning from the JD-R model and existing findings, we believe that job demands and resources at the beginning of teaching play a critical role in subsequent OWB. According to Tait (2008), beginning teachers with fewer job demands and more job resources would be more able to handle the adaptive changes and challenges during the transition period, which may facilitate the development of positive OWB in the long run, whereas if beginning teachers encounter a great deal of job demands but receive few resources, they may have difficulties in handling the changes and challenges. In addition, if this situation continues, they may feel overwhelmed with a reduced efficacy which may further cause disengagement and even attrition. In this sense, we believe that early levels of job demand and resources would predict the later OWB of beginning ECEs.

### The Current Research

In order to advance our understanding of the OWB of beginning ECEs, this study examines two questions: (1) what is the *status quo* of the OWB of Hong Kong beginning ECEs and (2) do job demands and resources predict later OWB? While the first question is exploratory in nature, we made a hypothesis for the second question. More specifically, we expected that beginning ECEs with fewer job demands and more job resources would have more positive OWB (i.e., higher levels of job satisfaction and work engagement while lower levels of job stress and job burnout). In addition, we expected that beginning ECEs with fewer job demands and more job resources would more likely be assigned as members of a more positive OWB profile. In this longitudinal study, we adopted both variable and person-centered approaches to examine the said questions, which may yield greater implications for how to enhance the OWB of beginning ECEs than the use of a single approach.

## Method

### Participants and Procedure

This study was part of a larger project that employed a three-wave longitudinal design to examine the extent to which individual factors (e.g., personality and mental health) during the training program would predict the job-related outcomes at work. At time 1 (T1), we collected data related to personality traits and mental health from students in their last semester of a 2-year, full-time early childhood education higher diploma program in the Education University of Hong Kong (*N* = 300, 96% females, covering 83.3% headcount of the cohort). By the time of the data collection, these students had completed qualifying internships and were expected to work in ECE sectors (e.g., kindergartens) upon graduation (not necessarily in the sectors where they did their internships). At the end of the first month of the Fall semester (time 2, T2), we followed up with the participants. Those who were working as in-service ECEs at the time of data collection were eligible to be followed up. A total of 213 participants responded to our invitation and 128 were eligible because they indicated that they were working as ECEs at the time. During the end of the fall semester and the beginning of the spring semester (time 3, T3), we again followed up with the participants. Eleven participants were excluded because they did not respond to our invitation, did not work as ECEs, or did not complete the questionnaires that measure the main variables, thus leaving 117 participants as the final sample (113 females, *M*_*age*_ = 21.71 years, SD = 3.09). Among the final sample, 111 participants were appointed as full-time kindergarten teachers and 6 as part-time kindergarten teachers, with 24 participants teaching at nursery class, 44 teaching at K1, 20 teaching at K2, and 29 teaching at K3. The average class size was 26.24 students (SD = 8.97) and the average daily working hours were 9.81 (SD = 1.46). Moreover, 21% of participants reported that they did not have SEN children in their class and 65.9% of participants earned more than HK$21,000 per month (US$1 = HK$7.8). The data reported in this study were from T2 and T3 because the questions in a study that were concerned about OWB and the prediction of job-related factors which were recruited were only at T2 and T3.

This study was reviewed and approved by the Human Research Ethical Committee of the Education University of Hong Kong (reference number: 2017-2018-0429). To collect data at T1, a research assistant approached the pre-service teachers in the classrooms and administered paper-and-pencil surveys during regular class hours. Participants were required to provide contact information (e.g., phone number and e-mail) for data matching purposes. To collect data at T2 and T3, the research assistant invited participants to join online surveys through e-mails, WhatsApp messages, and phone calls. Confidentiality was underscored across data collection and participants reserved the right to withdraw from each survey at any point. We selected the measurement moment for T1 according to the study schedule of pre-service teachers and the moments for T2 and T3 based on the study purpose (i.e., the prediction of job factors at the beginning of the semester on OWB one semester later). Participants received supermarket coupons with face values of HK$50, HK$100, and HK$150 upon completing the first, second, and third waves of the survey, respectively.

### Measures

#### Occupational Well-Being Indicators at T3

***Job satisfaction***. We employed the Chinese version of the Job Satisfaction Scale (Tsui et al., 1992) to measure how much participants were satisfied with their job. We adapted this measure to the current study by replacing the word “organization” in the original items with “kindergarten” to fit the working context of participants. This scale consists of six items rated on a five-point scale (from 1 = *strongly disagree* to 5 = *strongly agree*) and a higher mean score indicates higher job satisfaction. Sample item includes “Considering everything, how satisfied are you with your current job situation.” In this study, Cronbach’s alpha was 0.80.

***Work engagement***. We employed the Chinese version of Schaufeli et al. (2002) Work Engagement Scale (Wang et al., 2015) to measure the work engagement of participants. Although this scale was not specifically designed for educators, it has been widely used in various professions, including teachers (Hakanen et al., 2006; De Stasio et al., 2019). This scale consists of seventeen items divided into three dimensions: vigor, dedication, and absorption. The vigor dimension includes six items (e.g., “At my job I feel strong and vigorous”); the dedication dimension includes five items (e.g., “I am enthusiastic about my job”); and the absorption dimension includes six items (e.g., “When I am working, I forget everything else around me”). All items are rated on a five-point scale (from 1 = *strongly disagree* to 5 = *strongly agree*), and a higher mean score of each dimension indicates stronger work engagement. In this study, the Cronbach’s alpha was 0.79, 0.83, and 0.81 for the vigor, dedication, and absorption dimensions, respectively.

***Work stress***. We used the Teacher Stress Inventory (TSI; Fimian and Fastenau, 1990) to measure the work stress of participants. This measure was specifically designed to tap the stress of teachers. In this study, we translated the TSI into Chinese following a back-translation procedure (Van de Vijver and Hambleton, 1996). This scale lists sixteen stressors in the teaching context and participants were asked to indicate how stressed they feel for each stressor on a five-point scale (from 1 = *not stressful* to 5 = *highly stressful*). A higher mean score reflects more work stress. A sample item is “There is too much administrative paperwork in my job.” In this study, Cronbach’s alpha was 0.94.

***Job Burnout.*** We employed the Chinese version of the Maslach Burnout Inventory: Educators Survey (Yuen et al., 2002) to measure the job burnout of participants. This scale was specifically designed to measure the burnout of teachers at work. This scale consists of 22 items divided into three dimensions: emotional exhaustion, cynicism, and reduced efficacy. The emotional exhaustion dimension includes nine items (e.g., “I feel used up at the end of the workday”); the cynicism dimension includes five items (e.g., “I’ve become more callous toward people since I took this job”); and the reduced efficacy dimension includes eight items (e.g., “I deal very effectively with the problems of my students,” reverse scoring). All items are rated on a seven-point Likert-type rating scale (from 0 = *never* to 6 = *every day*). A higher mean score of each dimension indicates stronger burnout. In this study, the Cronbach’s alpha was 0.90, 0.84, and 0.85 for the emotional exhaustion, cynicism, and reduced efficacy dimensions, respectively.

#### Job Demands and Resources at T2

We measured job demands in terms of class size, working hours per week, and whether they had SEN children in the class (coded 1 = *Yes*, 2 = *No*). In addition, we measured job resources in terms of monthly salary and job support. To measure perceived support of participants from supervisors and colleagues, we employed the Job Support Scale (Skaalvik and Skaalvik, 2011). In this study, we translated this scale into Chinese following a back-translation procedure (Van de Vijver and Hambleton, 1996). This scale includes six items rated on a six-point scale (from 1 = *strongly disagree* to 6 = *strongly agree*). A higher mean score indicates that participants perceive more job support. A sample item includes “Teachers at this school help and support each other.” In this study, Cronbach’s alpha was 0.92.

### Data Analyses

We analyzed the data with SPSS 26.0 and Mplus 7.31 (Muthén and Muthén, 1998-2012). We first conducted attrition analyses by comparing job demands and job resources variables between those who provided complete data for this research (i.e., the complete group) and those who dropped at T3 (i.e., the attrition group). Regarding *variable-centered analyses*, we first conducted preliminary analyses (mean and standard deviations, and Pearson correlations). Then, we used multivariate regression (estimator = maximum likelihood; bootstrapping *N* = 5,000) to examine the extent to which T2 job demands and job resources factors predicted T3 OWB in Mplus. We simultaneously regressed all OWB indicators on job demands and resources to control for the concurrent covariance among OWB indicators. Subsequently, we used G^∗^Power 3.1.9.7 (F-test family, linear multiple regression R^2^ deviation from zero) to examine the sample size needed for 0.80 power with the obtained regression estimates. Regarding *person-centered analyses*, we first conducted LPA to explore OWB profiles using the robust maximum-likelihood estimator with robust standard errors (MLR), with scores of job satisfaction, vigor, dedication, absorption, job stress, emotional exhaustion, cynicism, and reduced efficacy as indicators. We tested the 1-profile model first, then the number of profiles was systematically increased until the best fitting model was identified. We determined the best-fitting model based on several model fit statistics: Akaike Information Criteria (AIC, Akaike, 1974), Bayesian Information Criterion (BIC, Schwarz, 1978), adjusted BIC (aBIC), Lo-Mendell-Rubin Adjusted Likelihood Ratio Test (LMRT, Lo et al., 2001), and Bootstrapped Likelihood-Ration Test (BLRT, Arminger et al., 1999). Regarding AIC, BIC, and aBIC, smaller values indicate better model fit. LMRT and BLRT evaluate the fit of a *k*-profile model (e.g., 3-profile model) to a *k*-1profile comparison model (e.g., 2-profile model), and the *p*-values associated with these statistics indicate whether the *k*-profile model (*p* < 0.05) or the *k*-1 profile model (*p* > 0.05) provides a better fit to the data. Moreover, the value of entropy no less than 0.6 indicates a good profile separation (Asparouhov and Muthén, 2014). In addition, we also considered the theoretical meaningfulness of the profiles (Nylund et al., 2007) and the proportion of participants represented in the profiles (Hipp and Bauer, 2006). A rule of thumb is that no profile should contain less than 5% of the respondents (Stanley et al., 2017). Given the modest sample size, we carried out Monte Carlo simulations to examine whether the current sample size was large enough to substantially distinguish the OWB profiles (Muthén and Muthén, 2002, Muthén and Muthén, 1998-2012). To this end, we used the current estimates and variance of each indicator in each profile as the starting values in combination with three sample sizes (*N* = 50, 80, and 110). The estimation processes were replicated 1000 times for each sample size. If sufficient power (i.e., 0.80 or above) was achieved with the given starting values and sample size, we deemed that our current sample size (>110) was adequate to achieve sufficient power. In the last step, we examined the association between T2 job demands and resources and T3 profile memberships with the R3STEP auxiliary command after identifying the best-fitting model (Asparouhov and Muthén, 2014). As the coefficients generated by the R3STEP function are logits, we exponentiated the logits into odd ratios for interpretation.

## Results

### Attrition Analyses

We used chi-square tests, independent t-tests, and Spearman correlations to examine the relationship between attrition group and T2 job demands and job resources. The results showed that the two groups did not significantly differ in whether there were SEN students in the class [χ^2^(1) = 0.03, *p* = 0.854], class size [*t*(126) = −0.62, *p* = 0.540], daily working hours [*t*(126) = 0.62, *p* = 0.537], or levels of job support [*t*(126) = −0.56, *p* = 0.576] at T2. Moreover, drop-out was also not significantly related to T2 salary (*ρ* = 0.06, *p* = 0.477). These findings suggested that our findings were not likely biased due to attrition.

### Variable-Centered Analyses

#### Preliminary Analyses

As summarized in Table 1, participants overall reported medium-to-high levels of job satisfaction (3.45 out of 5) and dedication (3.57 out of 5), medium levels of absorptive work engagement (3.24 out of 5), vigorous work engagement (2.99 out of 5), and emotional exhaustion (4.07 out of 7), low-to-medium levels of total job stress (2.71 out of 5), and low levels of reduced efficacy (2.59 out of 7) and cynicism (2.50 out of 7). Regarding bivariate associations, positive indicators were positively linked with one another, so were the negative indicators, and positive indicators were negatively related to negative indicators, except for four sets of correlations (i.e., job stress and absorption, job stress and vigor, absorption and cynicism, and emotional exhaustion and reduced efficacy). Finally, T2 job demand and job resources were related to T3 OWB indicators differently. Of note, T2 job support was related to all the positive and negative OWB indicators in the expected direction at statistically significant levels.

**TABLE 1:** Means, standard deviations, and bivariate correlations between occupational well-being (OWB) indicators for the total sample.

	***M***	***SD***	**1**	**2**	**3**	**4**	**5**	**6**	**7**	**8**	**9**	**10**	**11**	**12**
1. T2 Class size	26.24	8.97	−											
2. T2 SEN students in class (1 = yes, 2 = no)	1.20	0.40	0.27**	−										
3. T2 Working hours	9.81	1.46	0.12	0.05	−									
4. T2 Salary	5.24	1.26	0.09	–0.04	0.56***	−								
5. T2 Job support	4.42	0.82	–0.12	–0.10	–0.04	0.10	−							
6. T3 Job satisfaction	3.45	0.58	–0.08	0.01	−0.36***	–0.06	0.62***	−						
7. T3 Work engagement: absorption	3.24	0.64	0.02	0.01	–0.11	0.13	0.36***	0.41***	−					
8. T3 Work engagement: dedication	3.57	0.61	–0.06	0.12	−0.24**	0.11	0.42***	0.53***	0.68***	−				
9. T3 Work engagement: vigor	2.99	0.64	0.07	0.10	–0.15	0.06	0.37***	0.40***	0.77***	0.72***	−			
10. T3 Job stress	2.71	0.76	0.19*	–0.12	0.42***	0.22*	−0.26**	−0.50***	–0.09	−0.24**	–0.14	−		
11. T3 Job burnout: emotional exhaustion	4.07	1.31	0.14	−0.22*	0.44***	0.20*	−0.28**	−0.55***	−0.23**	−0.38***	−0.35***	0.63***	−	
12. T3 Job burnout: reduced efficacy	2.59	0.92	0.03	–0.08	0.06	–0.12	−0.32***	−0.34***	−0.35***	−0.48***	−0.34***	0.29***	0.14	−
13. T3 Job burnout: cynicism	2.50	1.43	0.08	–0.12	0.23*	0.03	−0.30**	−0.40***	–0.15	−0.34***	−0.26***	0.47***	0.59***	0.24**

*p < 0.05, **p < 0.01, ***p < 0.001.

#### Regression Analysis

Table 2 presents the predictive effects of T2 job demands and job resources on T3 OWB indicators. The model was saturated (i.e., chi-square = 0, RMSEA = 0, CFI and TLI = 1.00). It accounted for 48% variance of T3 job satisfaction, with longer working hours (*B* = −0.13, *SE* = 0.03, *p* < 0.001) negatively predicting this indicator and stronger job support (*B* = 0.42, *SE* = 0.05, *p* < 0.001) positively predicting the same indicator. The model accounted for 17% of the variance of T3 absorption, with higher salary (*B* = 0.10, *SE* = 0.05, *p* = 0.049) and stronger job support (*B* = 0.27, *SE* = 0.07, *p* < 0.001) positively predicting this indicator. The model accounted for 29% variance of T3 dedication, with longer working hours (*B* = −0.014, *SE* = 0.04, *p* = 0.001) negatively predicting and not having SEN students in class (*B* = 0.30, *SE* = 0.12, *p* = 0.011), higher salary (*B* = 0.13, *SE* = 0.05, *p* = 0.012), and stronger job support (*B* = 0.29, *SE* = 0.09, *p* = 0.001) positively predicting this indicator. The model accounted for 21% variance of T3 vigor, with longer working hours (*B* = −0.09, *SE* = 0, *p* < 0.042) negatively predicting and larger class size (*B* = 0.01, *SE* = 0.01, *p* = 0.028), not having SEN students in class (*B* = 0.31, *SE* = 0.15, *p* = 0.033), and stronger job support (*B* = 0.29, *SE* = 0.06, *p* < 0.001) positively predicting this indicator. The model accounted for 25% variance of T3 job stress, with longer working hours (*B* = 0.19, *SE* = 0.05, *p* < 0.001) positively predicting and stronger job support (*B* = −0.21, *SE* = 0.08, *p* = 0.009) negatively predicting this indicator. The model accounted for 31% variance of T3 emotional exhaustion, with longer working hours (*B* = 0.38, *SE* = 0.09, *p* < 0.001) positively predicting and not having SEN students in class (*B* = −0.80, *SE* = 0.25, *p* = 0.001) and stronger job support (*B* = −0.42, *SE* = 0.12, *p* = 0.001) negatively predicting this indicator. The model accounted for 14% variance of T3 reduced efficacy, with stronger job support (*B* = −0.35, *SE* = 0.13, *p* = 0.006) negatively predicting this indicator. The model accounted for 16% variance of T3 cynicism, with longer working hours (*B* = 0.25, *SE* = 0.10, *p* = 0.008) positively predicting and not having SEN students in class (*B* = −0.56, *SE* = 0.24, *p* = 0.021) and stronger job support (*B* = −0.49, *SE* = 0.18, *p* = 0.006) negatively predicting this indicator. Based on the standardized regression estimates presented in Table 2, we conducted power analyses in G^∗^Power. The results suggested that the required sample size needed for 0.80 power varied across OWB indicators, ranging from at least 21 participants needed for job satisfaction to 70 for reduced efficacy. Given that our current sample was 117, we considered that the current sample size would be sufficient to achieve 0.80 power for the regression results.

**TABLE 2:** Regression of OWB indicators on job demands and job resources.

	**T3 Job satisfaction**				**T3 Work engagement:**				**T3 Work engagement:**				**T3 Work engagement:**			
	**(*R*^2^ = 0.48)**				**absorption (*R*^2^ = 0.17)**				**dedication (*R*^2^ = 0.29)**				**vigor (*R*^2^ = 0.21)**			
	***B***	***SE***	***p***	**β**	***B***	***SE***	***p***	**β**	***B***	***SE***	***p***	**β**	***B***	***SE***	***p***	**β**
T2 Class size	0.00	0.01	0.570	0.04	0.01	0.01	0.360	0.09	0.00	0.01	0.461	0.06	0.01	0.01	0.028	0.18
T2 SEN	0.13	0.11	0.245	0.09	0.12	0.16	0.426	0.08	0.30	0.12	0.011	0.20	0.31	0.15	0.033	0.20
T2 Working hours	–0.13	0.03	<0.001	–0.34	–0.08	0.05	0.118	–0.19	–0.14	0.04	0.001	–0.34	–0.09	0.00	0.042	–0.20
T2 Salary	0.03	0.04	0.430	0.07	0.10	0.05	0.049	0.20	0.13	0.05	0.012	0.26	0.07	0.05	0.141	0.14
T2 Job support	0.42	0.05	<0.001	0.59	0.27	0.07	<0.001	0.34	0.29	0.09	0.001	0.39	0.29	0.06	<0.001	0.37

	**T3 Job stress**				**T3 Job burnout: emotional**				**T3 Job burnout: reduced**				**T3 Job burnout:**			
	**(*R*^2^ = 0.25)**				**exhaustion (*R*^2^ = 0.31)**				**efficacy (*R*^2^ = 0.14)**				**cynicism (*R*^2^ = 0.16)**			
	***B***	***SE***	***p***	**β**	***B***	***SE***	***p***	**β**	***B***	***SE***	***p***	**β**	***B***	***SE***	***p***	**β**

T2 Class size	0.01	0.01	0.252	0.08	–0.00	0.01	0.966	–0.00	–0.01	0.01	0.526	–0.05	–0.00	0.01	0.862	–0.01
T2 SEN	–0.22	0.17	0.183	–0.12	–0.80	0.25	0.001	–0.25	–0.31	0.18	0.080	–0.14	–0.56	0.24	0.021	–0.16
T2 Working hours	0.19	0.05	<0.001	0.37	0.38	0.09	<0.001	0.43	0.08	0.07	0.249	0.12	0.025	0.10	0.008	0.26
T2 Salary	0.02	0.06	0.680	0.04	–0.01	0.09	0.890	–0.01	–0.12	0.10	0.222	–0.17	–0.11	0.09	0.248	–0.10
T2 Job support	–0.21	0.08	0.009	–0.23	–0.42	0.12	0.001	–0.26	–0.35	0.13	0.006	–0.32	–0.49	0.18	0.006	–0.28

*SEN students in class (1 = yes, 2 = no).*

### Person-Centered Analyses

#### Occupational Well-Being Profiles Among Beginning Early Childhood Educators

As shown in Table 3, the results of LPA analysis suggested that the 2-profile solution described the optimal number of occupational well-being profiles. First, the 2-profile solution showed a better fit than the 1-profile solution, as indicated by significant LMRT (*p* = 0.023) and BLRT (*p* < 0.001) tests. By contrast, the 3-profile solution was not better than the 2-profile solution, as the LMRT test was not significant (*p* = 0.616). In addition, although the values of AIC, BIC, and aBIC decreased as the number of profiles increased, the magnitude of such decrease appeared more pronounced between the 1-profile and the 2-profile solutions than the one between the 2-profile and the 3-profile solutions. Moreover, for the 2-profile solution, no profile contained less than 5% of the respondents, whereas one of the profiles of the 3-profile solution contained less than 5% of the participants. Taken together, we selected the 2-profile solution as the final solution. This solution exhibited high entropy (i.e., 0.88) and the average posterior profile membership probability was high (i.e., 97.6% for the first and 95.4% for the second profile).

**TABLE 3:** Summary of latent profile models.

	**Log likelihood**	**Number of free Parameter**	**AIC**	**BIC**	**ABIC**	**Entropy**	**LMRT *p* value**	**BLRT *p* value**	**Class size per profile**
1 Profile	−1127.602	16	2287.205	2331.400	2280.882	–	–	–	117
**2 Profiles**	−**1020.995**	**25**	**2091.990**	**2161.045**	**2082.017**	**0.882**	**0.023**	**<0.001**	**83/34**
3 Profiles	−986.819	34	2041.638	2135.552	2028.075	0.930	0.616	<0.001	33/81/3

*Bolded entries represent the solution chosen in this study.*

Table 4 presents the raw scores of the OWB indicators for each profile and the results of multivariate analysis of variance (MANOVA). We labeled the first profile as “*medium well-being.*” This profile consisted of 71% of participants. A seeming characteristic of this profile is that the levels of all the positive indicators were above the mid-point (especially dedication), whereas the levels of all the negative indicators were below the mid-point (especially cynicism). Moreover, we labeled the second profile as “*relatively weak well-being – emotional exhaustion.*” This profile consisted of 29% of participants. The beginning ECEs assigned to this profile showed two characteristics: (1) the levels of all the positive indicators were a bit lower than, or close to, the mid-point and (2) the levels of stress were slightly higher than the mid-point, whereas the levels of exhaustion were seemingly above the mid-point. Of note, this group of participants also had relatively low reduced efficacy and cynicism.

**TABLE 4:** Means, standard deviations, and MANOVA tests for OWB indicators between the two profiles.

	**Profile 1: Medium**		**Profile 2: Relatively**		***F*(*1,115*)**	***p***	**Partial η^2^**
	**well-being**		**weak well-being – Emotional exhaustion**				
	**M**	**SD**	**M**	**SD**			
Job satisfaction	3.69	0.43	2.85	0.45	89.14	<0.001	0.44
Work engagement: absorption	3.47	0.50	2.70	0.62	49.81	<0.001	0.30
Work engagement: dedication	3.81	0.42	2.96	0.59	77.11	<0.001	0.40
Work engagement: vigor	3.23	0.51	2.42	0.55	57.14	0.001	0.33
Job stress	2.50	0.74	3.22	0.53	26.67	<0.001	0.19
Job burnout: emotional exhaustion	3.64	1.18	5.14	0.95	43.80	<0.001	0.28
Job burnout: reduced efficacy	2.29	0.73	3.33	0.92	42.16	<0.001	0.27
Job burnout: cynicism	2.05	1.20	3.58	1.36	36.36	<0.001	0.24

We conducted a MANOVA analysis to examine whether the levels of the OWB indicators were quantitively different between the two profiles. The results indicated a statistically significant difference in the multivariate test, Wilk’s λ = 0.338, *F*(8, 108) = 26.40, *p* < 0.001, partial η^2^ = 0.66. Follow-up examination of the between-subject effects revealed significant main effects. Compared to beginning ECEs assigned to the “*relatively weak well-being – emotional exhaustion*” profile, those assigned to the “*medium well-being*” profile reported significantly higher job satisfaction, absorption, dedication, and vigor, but lower job stress, emotional exhaustion, reduced efficacy, and cynicism.

#### Monte Carlo Analysis

The results of power analyses using Monte Carlo simulation found that with the current estimates as the starting values, the statistical power for each indicator and its variance ranged from 0.922 to 1.000, from 0.968 to 1.000, and from 0.986 to 1.000 when the number of observations was set to 50, 80, and 110, respectively. These statistical powers were larger than the conventional value (i.e., 0.80). Given that our sample size was 117, which was larger than 110, we deemed that our sample size was large enough to achieve adequate statistical power in distinguishing the two OWB profiles.

#### The Role of Job Demands and Resources in Occupational Well-Being Profiles

The results of the prediction of T2 job demands and resources on T3 OWB profiles are summarized in Table 5. As shown, all job demands and resources variables at T2 significantly predicted different OWB profiles at T3, except for class size. These findings suggested that relative to the “*relatively weak well-being – emotional exhaustion*” profile, one unit increase in working hours lowered the likelihood of being a member of the “*medium well-being*” profile by 0.35 times. Meanwhile, one unit increase in having no SEN students in the class, getting a higher salary, and receiving more job support increased the likelihood of being a member of the “*medium well-being*” profile by 10.70, 1.97, and 4.81 times, respectively. In sum, these findings indicated that Hong Kong beginning ECEs who reported fewer job demands (i.e., not having SEN students in the class and shorter working hours) and more job resources (i.e., higher salary and more job support) at the very beginning of teaching career will likely develop a more positive OWB profile later on.

**TABLE 5:** Logistic regression of class membership on predictors (with relatively weak well-being – emotional exhaustion as a reference group).

**Predictors**	**Estimate**	***SE***	***p***	**OR**	**OR 95% CI**
T2 Class size	−0.01	0.03	0.815	0.99	[0.93, 1.05]
T2 SEN students in class (1 = yes, 2 = no)	2.37	0.92	0.010	10.70	[1.76, 64.92]
T2 Working hours	−1.06	0.30	<0.001	0.35	[0.33, 0.37]
T2 Salary	0.68	0.34	0.046	1.97	[1.01, 3.84]
T2 Job support	1.57	0.46	0.001	4.81	[1.95, 11.84]

### Contrast Findings From the Variable-Centered and Person-Centered Approaches

Findings from the two approaches showed both similarity and uniqueness. For the similar part, results of the two approaches reconciled to reveal that fewer job demands and more job resources were generally beneficial to the OWB of beginning ECEs. For the unique part, results from the variable-centered approach disclosed how well the whole sample was doing on each OWB indicator, but such information did not allow us to assess whether there exist qualitative distinct subpopulations concerning OWB profiles. Given that the OWB construct consists of a series of indicators concerning varying aspects of it and, that beginning ECEs, do not score uniformly low or high on all indicators, the OWB profiles possess more abundant information on the attitude of beginning ECEs toward their work than a specific OWB indicator does. By contrast, results from the person-centered approach, which considered all the examined OWB indicators, identified two qualitatively distinct subgroups, which complemented the findings of the variable-centered approach. Besides this key uniqueness, some other uniqueness included that the variable-centered approach demonstrated the varying magnitude of the prediction of job demands and/or job resources on each OWB indicator. This information may inform which specific job demand or resources could be targeted at to enhance a particular OWB indicator. By contrast, the predictive pattern of the person-centered approach suggested that how likely a specific job demand or resource might distinguish different subgroups, thereby providing information that helps identify vulnerable subgroups who might be in more dire need of support.

## Discussion

Given the importance of the OWB of ECEs in a range of job-related outcomes and the multiple changes and challenges during the transition from training to teaching, there is a dire need to understand the OWB of beginning ECEs. This study employed both variable-centered and person-centered approaches to explore the OWB of Hong Kong beginning ECEs and examined the role of job demands and resources. The variable-centered analysis found that beginning ECEs showed medium and above-medium levels of positive OWB indicators and medium and low levels of negative OWB indicators. The person-centered analysis further identified that beginning ECEs with different levels of OWB indicators could be categorized into two distinct OWB profiles: the “*medium well-being*” and the “*relatively weak well-being – emotional exhaustion.*” Moreover, confirming our hypothesis, both variable-centered and person-centered results showed that fewer job demands and more job resources were associated with more positive OWB. To the best of our knowledge, this research is among the first to adopt both variable-centered and person-centered approaches to examine the OWB of beginning ECEs, especially in the Hong Kong context.

### Occupational Well-Being of Beginning Early Childhood Educators

Previous findings suggested that compared to junior teachers, veteran teachers were not only more satisfied with their job (especially salary), but they also showed higher levels of burnout symptoms and less aspiration (Wong and Zhang, 2014; Chaaban and Du, 2017; Jones et al., 2019; Li et al., 2020; Schaack et al., 2020). By contrast, current results of the variable-centered approach suggested that the levels of the dedication of Hong Kong beginning ECEs were above medium and job satisfaction were medium, whereas the levels of two types of job burnout symptoms (i.e., reduced efficacy and cynicism) were low. These findings were somewhat consistent with the aforementioned findings from veteran teachers. However, since there were a number of OWB indicators and the level of each indicator was varied, only relying on the findings from the variable-centered approach did not allow us to qualitatively determine how good the OWB of the current sample was. With a person-centered approach that simultaneously considered all the examined indicators, we were able to reveal two qualitatively distinct subgroups (i.e., medium well-being and relatively weak well-being – emotional exhaustion), which complemented the findings from the variable-centered approach.

The results of the person-centered approach yielded some unique information. First, the results qualitatively showed that our current sample did not experience absolutely negative or absolutely positive OWB as no such profiles were identified. Second, about one-third of participants showed obvious emotional exhaustion during the early stage of teaching (i.e., the “*relatively weak well-being – emotional exhaustion*” profile). In other words, although many beginning ECEs in our sample did not experience alarming negative OWB issues, a proportion of them showed symptoms of emotional exhaustion at the early stage of their teaching career. Given that the core ideas of positive psychology suggest that optimal well-being should be reflected in the absence of ill-being and in the presence of well-being (Seligman and Csikszentmihalyi, 2000), we reckon that the OWB of the current sample is not optimal and needs to be strengthened, especially in mitigating their emotional exhaustion and enhancing the positive aspects of OWB.

Schaufeli et al. (2006) suggested that positive and negative OWB indicators are seemingly contradictory and that they are supposed to be adversely related to one another. Prior variable-centered studies found that ECEs with high levels of positive OWB indicators tend to score low on negative indicators, and vice versa (Yeşil Dağli, 2012; Gu et al., 2020; Penttinen et al., 2020; Li et al., 2021). The current results of the bivariate correlation analyses were largely, but not entirely, consistent with the aforementioned studies as some sets of bivariate correlations were not significant. On one hand, this implies that beginning ECEs high in some positive OWB indicators do not necessarily report low levels on some negative OWB indicators (and vice versa). On the other hand, it suggests the need to use a person-centered approach to conduct a more nuanced examination on the configuration of different OWB indicators in beginning ECEs. Noticeably, the current results are consistent with prior research which suggests that employees may simultaneously endorse seemingly contradictory OWB indicators (Moeller et al., 2018; Salmela-Aro et al., 2019). This also highlights the merits of applying LPA to investigate job-related attitudes and behavior of employees in an organizational setting (Spurk et al., 2020).

### The Role of Job Demands and Job Resources

Confirming our hypotheses, both approaches revealed that fewer job demands and more job resources were related to better OWB. These results were largely consistent with previous variable-centered findings (Watt and Richardson, 2008; Yang and Zhao, 2016; Zinsser et al., 2016; Li and Li, 2020; Schaack et al., 2020) and supported the JD-R model (Bakker and Demerouti, 2014). Despite the similarity of the findings from both approaches, they provided different information and suggestions. For the variable-centered approach, job demands and job resources examined in this study predicted the OWB indicators differently. For instance, class size only predicted vigor while salary only predicted absorption and dedication. These findings informed *which* specific job demands and resources should be targeted to enhance a particular OWB indicator. For the person-centered approach, the predictive pattern, as mentioned earlier in the result section, informed how likely the examined job demands and resources could be used to identify subgroups *who* are more vulnerable and need more support to enhance their well-being at work. It is worthwhile to note that our study only examined a few job demands and resources relevant to ECEs, and most of them were demographics-oriented. Thus, it would be promising for future research to conduct a more systematic investigation by including other job demands (e.g., emotional demands and workload demands) and job resources (e.g., job control and leadership) relevant to the early childhood education setting to reveal a more complete picture of the risk and protective factors of the OWB of beginning ECEs.

### Implications

The current findings have some implications for the examination and enhancement of the OWB of beginning ECEs. First, although few alarming negative OWB issues were found in the current sample, about one-third of them showed emotional exhaustion at the very early stage of their teaching career. In addition, no absolute positive profile of OWB was identified. These findings highlighted the importance to reveal the subgroups who experienced emotional exhaustion and mitigate this issue among them and enhance the positive OWB indicators among the overall population. Second, as fewer job demands and more job resources at the early stage of teaching are related to a subsequently more positive OWB profile, it would be important for organizations to establish a facilitative working environment and guidance to promote adaptation and OWB of beginning ECEs. Finally, as beginning ECEs do not enter the workplace as a blank state but with their own abilities and characters (Tait, 2008), it is therefore important for ECE training institutes to provide pre-service teachers with systematic training to enhance competence and capacities to cope with job-related challenges and adaptive changes of their students so that they can develop a positive OWB during the transition from school to work. Findings from the variable-centered approach may also inform what specific predictors could be targeted.

### Limitations and Future Directions

We must acknowledge that this study has several limitations. First, our current study focused on OWB among beginning ECEs who graduated from a two-year higher diploma program and worked for one semester in Hong Kong kindergartens. Given the differences between higher diploma and bachelor programs in terms of length and depth of training, the current findings may not be fully generalized to graduates from bachelor or other training programs. Relatedly, this research was conducted in Hong Kong, where education competition is fierce and the aspiration of parents for success by their children is high (Wong and Rao, 2015), and, therefore, the generalizability of the findings to other cultural contexts may be limited. It is important for future research to revisit this issue in other regions, especially from a cross-cultural perspective. Moreover, readers should be cautious that the current findings were mainly drawn from female ECEs as the gender ratio in this study was far from balance (i.e., 113 women vs. 4 men), although this ratio is quite consistent with the government census (Education Bureau, 2021). We encourage future studies to address this issue by including more male ECEs. Second, our sample size was relatively small, although the results of Monte Carlo analyses indicated sufficient statistical power for LPA. Nevertheless, it would be desirable for future research to use a larger sample size to re-examine this issue to obtain a more conclusive finding. Third, although we collected data at different time points as the study was correlational in nature and these data preclude strong claims regarding a causal influence of job demands and resources on OWB. Of note, we did not control for the baseline levels of OWB at T2 as the main research question in this study was about the relationships between the individual differences in T2 job demands or resources and T3 OWB rather than how T2 job demands or resources predicts the changes in OWB over time. It would be promising for future research to measure OWB indicators repeatedly so that their trajectories could be examined. Relatedly, the JD-R model also suggests that job demands and job resources may be reciprocally related to OWB indicators (Bakker and Demerouti, 2014). Future research may consider investigating this issue among beginning ECEs by following up with them for a certain period, as the findings would inform how the OWB of beginning ECEs changes over time and would disclose the course from entering to quitting the profession or sector. Moreover, future research may also consider carrying out intervention programs to examine whether manipulating job demands and resources would lead to corresponding changes in subsequent OWB.

### Conclusion

The OWB of Beginning ECEs is important to job performance, child development, and the operation of early childhood education sectors. Using both variable and person-centered approaches, we find that the levels of the OWB indicators of Hong Kong beginning ECEs vary and that the complex pattern of these indicators could be categorized into two distinctive profiles (i.e., *medium well-being* and *relatively weak well-being – emotional exhaustion*). Moreover, we also disclose that fewer job demands and more resources upon employment are important for beginning ECEs to develop positive OWB subsequently. These results inform *which* leverage points could be targeted to enhance a specific OWB indicator and identify *who* would be in dire need to enhance their OWB at the very beginning of their teaching career.

## Data Availability Statement

The raw data supporting the conclusions of this article will be made available by the authors, without undue reservation.

## Ethics Statement

The studies involving human participants were reviewed and approved by The Education University of Hong Kong. The patients/participants provided their written informed consent to participate in this study.

## Author Contributions

J-BL and AY contributed to conception and designed of the study. J-BL and TL collected the data. J-BL, AY, and RZ performed the statistical analysis. J-BL wrote the first draft of the manuscript. AY, RZ, TL, and ZL wrote sections of the manuscript. All authors contributed to manuscript revision, read, and approved the submitted version.

## Conflict of Interest

The authors declare that the research was conducted in the absence of any commercial or financial relationships that could be construed as a potential conflict of interest.

## Publisher’s Note

All claims expressed in this article are solely those of the authors and do not necessarily represent those of their affiliated organizations, or those of the publisher, the editors and the reviewers. Any product that may be evaluated in this article, or claim that may be made by its manufacturer, is not guaranteed or endorsed by the publisher.
